# Was muss der Allgemein- und Viszeralchirurg von der onkologisch ausgerichteten Strahlentherapie wissen?

**DOI:** 10.1007/s00104-023-01820-1

**Published:** 2023-03-09

**Authors:** Jörg Andreas Müller, Simon Trommer, Frank Meyer, Katharina Lampe, Roland S. Croner, Dirk Vordermark, Daniel Medenwald

**Affiliations:** 1 Universitätsklinik und Poliklinik für Strahlentherapie, Universitätsklinikum Halle (Saale), Halle (Saale), Deutschland; 2 Klinik für Allgemein‑, Viszeral‑, Gefäß- und Transplantationschirurgie, Universitätsklinikum Magdeburg A. ö. R., Leipziger Str. 44, 39120 Magdeburg, Deutschland; 3 Klinik für Strahlentherapie, Universitätsklinikum Magdeburg A. ö. R., Magdeburg, Deutschland

**Keywords:** Multimodale Tumortherapiekonzepte, Rektumkarzinom, Ösophaguskarzinom, Analkarzinom, Lebermetastasen, Multimodal tumor treatment concepts, Rectal cancer, Esophageal cancer, Anal cancer, Liver metastases

## Abstract

**Hintergrund:**

Die Strahlentherapie ist ein integraler Bestandteil in den meisten modernen multimodalen Tumortherapiekonzepten sowohl in kurativen als auch in palliativen Therapiesituationen. Dies gilt auch für viele Tumorentitäten im allgemein- und viszeralchirurgischen Bereich. Dabei kann es zu neuen Herausforderungen im Rahmen des klinischen Alltags und der interdisziplinären Tumorkonferenzen kommen.

**Ziel:**

Praxisrelevanter Überblick, basierend auf selektiven Referenzen der aktuellen medizinisch-wissenschaftlichen Literatur und gewonnenen klinischen Alltagserfahrungen, für den onkologisch tätigen Chirurgen über strahlentherapeutische Therapieoptionen bei viszeralmedizinischen Tumoren mit dem Fokus auf die viszeralonkologischen Tumoren wie Rektumkarzinom, Ösophaguskarzinom, Analkarzinom und Lebermetastasen

**Methode:**

Es wird eine narrative Übersicht präsentiert.

**Ergebnisse (selektive Eckpunkte):**

In ausgewählten Fällen ist es beispielsweise möglich, dass im Rahmen neuer Konzepte beim Rektumkarzinom ein derart gutes Ansprechen erreicht wird, dass unter engmaschiger Kontrolle eine Resektion vermieden werden kann. Beim Ösophaguskarzinom gilt die neoadjuvante Radiochemotherapie mit anschließender Resektion bei allen geeigneten Patienten als Therapieregime der Wahl. Sollte eine Operation nicht infrage kommen, so steht mit der definitiven Radiochemotherapie, insbesondere bei einem Plattenepithelkarzinom, eine gute Alternative zur Verfügung. Beim Analkarzinom bleibt auch im Licht neuster Erkenntnisse die primär definitive Radiochemotherapie als strahlentherapeutische Therapie der Wahl. Lebertumoren können mithilfe der stereotaktischen Strahlentherapie lokal abladiert werden. Mit der Leberstereotaxie ist ein hoch wirksames Mittel mit geringer Nebenwirkungsrate verfügbar.

**Schlussfolgerung:**

Auch vor dem Hintergrund der jüngsten Studien bleibt die enge Zusammenarbeit der Disziplinen im Rahmen der Tumortherapie essenziell zur bestmöglichen Therapie der betroffenen Patienten.

## Abstract (en)

**Background:**

Radiotherapy is an integral component of most modern multimodal tumor treatment concepts, both in palliative and curative situations and intentions. This also applies to many tumor entities relevant in general as well as abdominal surgery. This can give rise to new challenges in the context of the daily clinical routine and interdisciplinary tumor conferences.

**Aim:**

Practice relevant overview, based on selective references from the current scientific literature in medicine and own experiences obtained in daily work, for the oncological surgeon on radiotherapy-associated options for visceral tumor lesions. A particular focus is on rectal cancer, esophageal cancer, anal cancer and liver metastases.

**Method:**

A narrative review is given.

**Results (selected corner points):**

In total neoadjuvant therapy it is possible to avoid resection in rectal cancer if a good response is achieved and close monitoring can be provided. In esophageal cancer neoadjuvant chemoradiotherapy followed by resection can be considered the therapeutic regimen of choice for all suitable patients. If surgery is not an option, definitive chemoradiotherapy is an appropriate and favorable alternative, especially with respect to squamous cell carcinoma. Even taking the latest data on the topic into account, definitive chemoradiotherapy remains undisputedly recommended for anal cancer. Liver tumors can be locally ablated by stereotactic radiotherapy.

**Conclusion:**

Close cooperation between disciplines in the context of tumor therapy remains essential for the best possible treatment and outcome of patients.

## Hintergrund

Der Fachbereich der Strahlentherapie zeichnet sich durch ein hohes Maß an Interdisziplinarität aus. In enger Kooperation mit den Kollegen der anderen onkologischen Fachbereiche wird in den Tumorboards die bestmögliche, insbesondere befundbezogene und patientenindividuelle Diagnostik und Therapie für die Patienten geplant und durchgeführt. Für diesen interdisziplinären Austausch ist ein grundlegendes Verständnis der Therapien der einzelnen Fachbereiche vorteilhaft.

In dieser Übersichtsarbeit wird ein praxisrelevanter Überblick für den onkologisch tätigen Chirurgen über die Entwicklung und den etablierten Stand strahlentherapeutischer Therapieoptionen bei viszeralmedizinischen Tumoren gegeben mit dem Fokus auf die wichtigsten Entitäten viszeralonkologischer Tumoren wie dem Rektumkarzinom, dem Ösophaguskarzinom, dem Analkarzinom sowie auf die Stereotaxie von Lebermetastasen. An geeigneter Stelle wird auch auf radioonkologische Aspekte bildgebender Verfahren eingegangen. Es wurden wichtige Studien der letzten Jahre ausgewählt, die einen entscheidenden Einfluss auf die Erarbeitung der aktuellen Leitlinien hatten oder in Zukunft haben könnten.

## Methode

Es wird eine narrative Übersicht basierend auf selektiven Referenzen der aktuellen medizinisch-wissenschaftlichen Literatur und gewonnenen klinischen Alltagserfahrungen gegeben.

## Ergebnisse

### Ösophaguskarzinom

#### Epidemiologie

Bei den Malignomen der Speiseröhre lassen sich als Hauptformen das Plattenepithel- und das Adenokarzinom identifizieren.

Das mediane Erkrankungsalter beträgt bei Männern für beide Formen des Ösophaguskarzinoms 67 Jahre, Frauen erkranken mit 70 Jahren (Plattenepithelkarzinom) bzw. 73 Jahren (Adenokarzinom) einige Jahre später. Das relative 5‑Jahres-Überleben liegt für das Adenokarzinom bei 29 % und für das Plattenepithelkarzinom bei 17 % [1].

#### Therapie

Entsprechend den Empfehlungen der als „Living Guideline“ konzipierten S3-Leitlinien (Tab. 1) zur Behandlung des Ösophaguskarzinoms wird bei einem lokal fortgeschrittenen Adenokarzinom des Ösophagus oder des ösophagogastralen Übergangs (Kategorie cT3/T4 resektabel oder Kategorie cN1-3) eine perioperative Chemotherapie oder präoperative Radiochemotherapie empfohlen.

Weiterhin soll entsprechend den Empfehlungen der Guideline beim operablen Patienten mit lokal fortgeschrittenem Plattenepithelkarzinom des Ösophagus (Kategorie cT3/T4 resektabel oder Kategorie cN1-3) in jedem Fall eine präoperative Radiochemotherapie mit anschließender kompletter Resektion angestrebt werden.

**Table 1:** 

		Plattenepithelkarzinome	Adenokarzinome
Neoadjuvanz	Chemotherapie	Keine Empfehlung	Durchführung kann bei cT2-Tumoren Ösophagus + ösophagogastraler Übergang erwogen werden. Postoperative Fortsetzung empfohlen
	Radiatio	Kann beim operablen Patienten mit einem resektablen Ösophaguskarzinom nicht empfohlen werden	
	Radiochemotherapie	Bei operablen cT2-Tumoren kann Therapie erwogen werden Durchführung bei operablen cT3/cT4-Tumoren empfohlen	Durchführung bei operablen cT3/cT4-Tumoren Ösophagus + ösophagogastraler Übergang empfohlen
Adjuvanz	Chemotherapie	Keine Empfehlung	Nach R0-Resektion nicht empfohlen
	Radiatio	Nach R0-Resektion nicht	Keine Empfehlung
	Radiochemotherapie	Empfohlen	Kann bei Tumoren des ösophagogastralen Übergangs nach R0-Resektion mit erhöhtem Lokalrezidivrisiko bei nicht neoadjuvant vorbehandelten Patienten durchgeführt werden
Primar definitve Radiochemotherapie	–	Soll unabhängig von Histologie bei nichtresektablen Tumoren oder funktionell inoperablen Patienten durchgeführt werden	
Primar definitve Radiochemotherapie vs. primäre Resektion	Zervikales Ösophaguskarzinom	Lokalisiert zervikale Tumoren sollten primär mit einer definitiven Radiochemotherapie behandelt werden	Keine Studien vorhanden
	Thorakales Ösophaguskarzinom	Bei resektablen cT3/cT4-Tumoren kann alternativ zur Resektion eine definitive Radiochemotherapie erfolgen	Keine Studien vorhanden

#### Neoadjuvante Radiochemotherapie

In der niederländischen CROSS-Studie (Tab. 2) wurden 368 Patienten mit einem operablen Ösophaguskarzinom oder einem Adenokarzinom des ösophagogastralen Übergangs (AEG) mit den TNM-Stadien T1N1 oder T2-3N0‑1 in zwei Studienarmen verglichen. In der einen Gruppe wurden die Patienten lediglich in kurativer Intention operiert; die andere Gruppe wurde zusätzlich neoadjuvant mit 41,4 Gy bestrahlt und erhielt simultan eine Chemotherapie mit Carboplatin AUC2 sowie Taxol 50 mg/m^2^ Körperoberfläche (KOF) bis zu 5 × in wöchentlichen Gaben.

**Table 2:** 

Studie	Einschlusskriterien (Auswahl)	Wesentliche Ergebnisse
CROSS	Stadien T1N1 oder T2‑3 N0‑1 75 % Adenokarzinom *n* = 368 OP ± Radiotherapie 41,4 Gy und Carboplatin AUC2 + Taxol 50 wöchentlich (× 5)	R0-Resektion: im Arm „nur OP“: 69 % im Arm „RCHT + OP“: 92 % Pathologische Komplettremission nach Radiochemo: 29 % OP-assoziierte Todesfälle: jeweils 4 % im Arm „nur OP“ und im Arm „RCHT + OP“
ARTDECO	*n* = 260 Phase-III-Studie zur Dosisintensität der Radiochemotherapie: 50,4 Gy (Standard) vs. 61,6 Gy (eskaliert; simultan integrierter Boost auf den Primärtumor, nicht auf pathologische Lymphknoten), Stadien cT1‑4 N0‑3 M0 (M1 supraklavikulär) Plattenepithel-CA oder Adeno-CA Carboplatin AUC2 + Taxol 50 wöchentlich (× 5)	Grad-5-Toxizität: 3,3 % im Standarddosis-Arm und 7,6 % im Hochdosis-Arm (Blutung, Fistel, Ateminsuffizienz, Sepsis) Die Dosis von 50,4 Gy wird von den Autoren als Standard angesehen, Therapieintensivierung eher über neue Substanzen

*CA* Karzinom, *OP* Operation, *RCHT* Radiochemotherapie

Die Ergebnisse der Studie zeigten einen klaren Vorteil für die Gruppe der neoadjuvant vorbehandelten Patienten. Eine R0-Resektion war bei dieser Gruppe zu einem deutlich höheren Prozentsatz möglich (69 % im Arm „nur Operation“ vs. 92 % im Arm „RCHT (Radiochemotherapie) + Operation“; *p* < 0,001). Zudem zeigte sich im Gesamtüberleben der Patienten bereits nach 3 Jahren Beobachtungszeitraum ein signifikanter Vorteil für die neoadjuvant vorbehandelte Gruppe. Dieser Überlebensvorteil konnte für alle histologischen Typen gezeigt werden [2].

Im Jahr 2021 wurden die Langzeitdaten der CROSS-Studie veröffentlicht. Unter anderem konnte der Vorteil bezüglich des Gesamtüberlebens der neoadjuvant vorbehandelten Gruppe erneut gezeigt werden (medianes 12-Jahres-Gesamtüberleben: 38 % vs. 25 %, *p* = 0,004). Zudem reduzierte die neoadjuvante Radiochemotherapie das Risiko lokoregionärer Rezidive [3].

#### Definitive Radiochemotherapie

**Figure 1:**
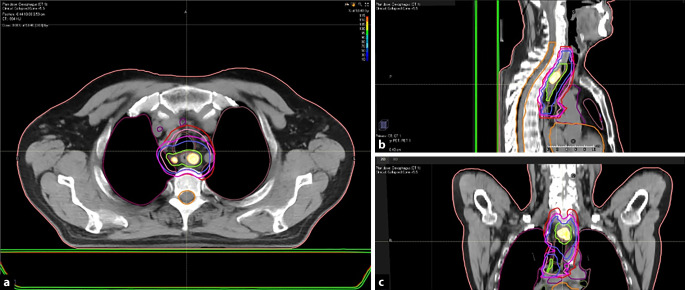

Gemäß der S3-Leitlinie wird eine definitive Radiochemotherapie unabhängig von der histologischen Entität empfohlen, wenn der Tumor nicht resektabel bzw. der Patient funktionell inoperabel ist oder der Patient die Operation ablehnt (Abb. 1).

Zudem kann laut den Empfehlungen der Leitlinienkommission bei einem resektablen Plattenepithelkarzinom der Kategorie cT3/cT4 oder bei positiven thorakalen Lymphknoten alternativ zur Resektion eine definitive Radiochemotherapie erwogen werden.

Erstmals konnte 1992 bei Patienten mit einem lokal fortgeschrittenen Plattenepithel- oder Adenokarzinom des thorakalen Ösophagus (Stadien cT3‑4 N0‑1 M0) ein Vorteil der Radiochemotherapie gegenüber der alleinigen Radiatio (RTX) gezeigt werden (Gesamtüberleben RCHTH-Gruppe: 26 % vs. alleinige RTX: 0 %, *p* < 0,001) [4].

Mit der Frage der Intensität der definitiven Radiochemotherapie beschäftigte sich der ARTDECO-Trial (Tab. 2). In dieser Phase-III-Studie erhielt eine Gruppe die Standarddosis von 50,4 Gy, während die andere Gruppe mit einem eskalierten Dosiskonzept von 61,6 Gy therapiert wurde. Beide Gruppen wurden mit einer simultanen Chemotherapie mit Carboplatin und Paclitaxel behandelt und erhielten eine obligatorische PET(Positronenemissionstomographie)-Untersuchung für die Planung der Bestrahlung. Die Stadien cT1‑4 N0‑3 M0 (M1 supraklavikulär) wurden in die Studie eingeschlossen.

Bezüglich des primären Endpunktes der lokalen Kontrolle der Tumoren konnte kein signifikanter Unterschied zwischen beiden Studienarmen gezeigt werden. Darüber hinaus überwogen tumorbedingte Todesfälle (u. a. Blutungen, Fistelungen, Ateminsuffizienz, Sepsis) im Hochdosis-Arm mit 7,6 % gegenüber 3 % im Standarddosis-Arm (*p*-Wert zeigte keinen signifikanten Unterschied an). Die Autoren empfahlen, auf Grundlage dieser Ergebnisse die Dosis von 50,4 Gy als Standard anzusehen und eine weitere Therapieintensivierung eher über ergänzende Therapeutika zu erzielen [5]. Häufig wird, wie an der berichtenden Klinik, vor dem Hintergrund dieser Daten ein Kompromiss geschlossen und eine lokale Boostbestrahlung, also eine Aufsättigung nur der PET-positiven Areale mit 1,8 ad 5,4 Gy nach der Hauptserie mit 50,4 Gy durchgeführt.

#### Vergleich neoadjuvante Radiochemotherapie und Resektion vs. primär definitive Radiochemotherapie

Eine randomisierte Studie beim lokal fortgeschrittenen Plattenepithelkarzinom des Ösophagus (Stadien cT3‑4 N0‑1 M0) verglich die primär definitive Radiochemotherapie mit der neoadjuvanten Radiochemotherapie und anschließender Operation. Hierbei konnte im Operations-Arm eine R0-Resektion in 82 % der Fälle erreicht werden; eine pathologische Komplettremission zeigte sich in 35 % der Fälle. Bezüglich des 10-Jahres-Überlebens wurde in dieser Studie ein Vorteil für die neoadjuvante Vorbehandlung mit anschließender Operation gezeigt (Gesamtüberleben Radiochemotherapie + Operation: 19 % vs. 12 % alleinige Radiochemotherapie, *p*-Wert zeigte keinen signifikanten Unterschied an; [6]).

In einer Metaanalyse wurde die Präferenz für die trimodale Therapie gegenüber der bimodalen Radiochemotherapie ausgesprochen, da in allen dort eingeschlossenen Studien ein Überlebensvorteil für die zusätzlich operierte Gruppe gezeigt werden konnte (Hazard Ratio [HR] aller eingeschlossenen Studien: 0,55 [0,49; 0,62]; [7]).

Aktuell laufende Studien wie die ESOPEC- und die RACE-Studie evaluieren weitere Optimierungsmöglichkeiten der multimodalen Therapie des Ösophaguskarzinoms. Im Rahmen dieser Studien soll geprüft werden, ob die vor und nach der Operation gegebene Chemotherapie (5-Fluorouracil, Leucovorin, Oxaliplatin, Docetaxel [FLOT]) wirksamer als die kombinierte neoadjuvante Radiochemotherapie nach dem CROSS-Protokoll ist. Es bleibt abzuwarten, welchen Einfluss die Ergebnisse dieser Studien auf die zukünftige Therapie des Ösophaguskarzinoms haben werden.

#### Schlussfolgerung

Bei resektablen Ösophaguskarzinomen beider histologischer Subtypen sollte die neoadjuvante Radiochemotherapie mit anschließender Resektion der alleinigen Radiochemotherapie vorgezogen werden. Durch diese Vorbehandlung kann sowohl die R0-Resektabilität als auch das Gesamtüberleben verbessert werden. Bei inoperablen Ösophaguskarzinomen wird unabhängig vom histologischen Subtyp eine definitive Radiochemotherapie empfohlen. Eine Dosiseskalation über die Standarddosis hinaus führt nicht zu einem verbesserten Outcome.

### Rektumkarzinom

#### Epidemiologie

Mit einer Inzidenz von 28,8 bis 32,1 pro 100.000 Einwohner in Europa zählt das kolorektale Karzinom zu den häufigeren Tumoren. Weltweit stellt das kolorektale Karzinom das dritthäufigste Malignom dar. In bis zu 95 % der Fälle liegt dabei ein Adenokarzinom vor. 28 % der kolorektalen Karzinome sind im Rektum lokalisiert [8, 9].

#### Neoadjuvanter Therapieansatz

**Figure 2:**
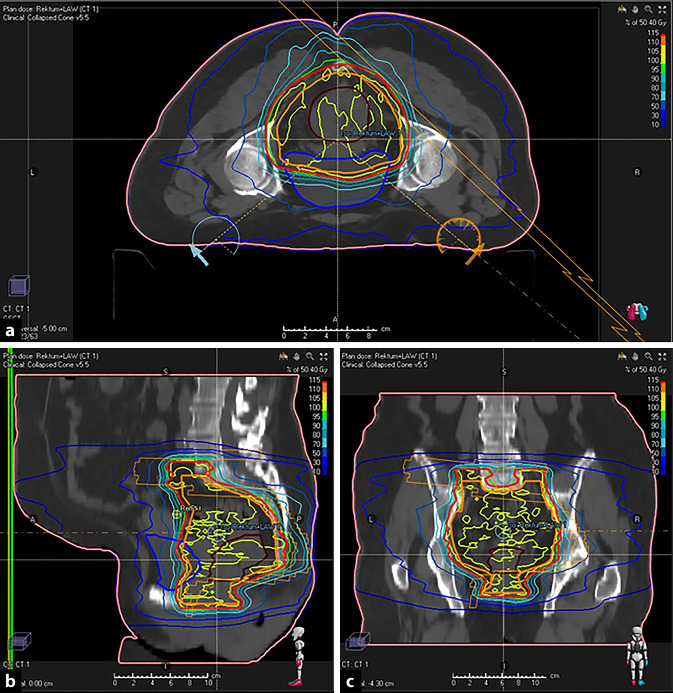

In der Therapie des Rektumkarzinoms können zwei in der aktuellen S3-Leitlinie von 2019 verankerte Konzepte als Standardtherapie betrachtet werden: Einerseits die neoadjuvante Radiatio mit 5 mal 5 Gy und unmittelbar anschließender Resektion als Kurzzeitkonzept, andererseits die neoadjuvante Radiochemotherapie mit 1,8 ad 50,4 Gy und simultaner Gabe einer fluorouracilhaltigen Chemotherapie, gefolgt von einer Resektion nach einem Intervall von 4 bis 8 Wochen (Abb. 2). Dieses Vorgehen gründet sich im Wesentlichen auf je drei Studien zum Kurzzeitkonzept [10–12] und zur Radiochemotherapie [13–15]. Hierbei wird der Radiochemotherapie in der Regel insbesondere bei T3-Tumoren, tiefliegenden Tumoren mit intendiertem Sphinktererhalt sowie bei Nähe des Tumors zur mesorektalen Faszie der Vorzug gegeben.

In zwei Studien wurden beide Konzepte miteinander verglichen. Hierbei zeigte sich zusammenfassend eine höhere Akuttoxizität, aber auch eine reduzierte Rate an Resektionen mit positivem Resektionsrand im Rahmen der simultanen Radiochemotherapie. Der Sphinktererhalt, das Gesamtüberleben und die Spättoxizität waren nach beiden Therapien vergleichbar [16–18]. In einer Studie zeigte sich eine Tendenz zu einer niedrigeren Lokalrezidivrate nach simultaner Radiochemotherapie, dieser Unterschied erreichte jedoch keine statistische Signifikanz [18]. Während sich durch beide Konzepte in Kombination mit einer TME eine gute lokale Kontrolle erreichen lässt, bleibt das Gesamtüberleben jedoch unverändert [12, 19]. Dies könnte darin begründet sein, dass das Auftreten von Fernmetastasen durch eine prä- vs. einer postoperativen Therapie nicht signifikant beeinflusst wird [19].

#### Neoadjuvantes Kurzzeitkonzept 5 × 5 Gy

Die Stockholm-III-Studie (Tab. 3) adressierte die Frage, inwiefern nach erfolgter neoadjuvanter Kurzzeitradiotherapie mit 5 × 5 Gy eine Resektion auch im Intervall erfolgen kann. In der Kontrollgruppe wurde eine Woche nach erfolgter Radiatio mit 5 × 5 Gy die tiefe anteriore Resektion (TAR) mit totaler mesorektaler Exzision (TME) durchgeführt und mit einer Resektion nach 4 bis 8 Wochen nach Radiatio mit 5 × 5 Gy verglichen. Der primäre Endpunkt dieser Nichtunterlegenheitsstudie war die Zeit bis zum Lokalrezidiv. Im Rahmen der Studie konnte die Äquivalenz beider Konzepte sowohl hinsichtlich der Zeit bis zum Lokalrezidiv als auch der Entstehung einer Fernmetastasierung gezeigt werden [20]. Weiterhin war die Rate an Komplettremissionen bei verzögerter TAR mit TME signifikant erhöht (10,1 % vs. 1,7 %; *p* < 0,001), gleichzeitig manifestierten sich nach verzögerter TAR mit TME häufiger behandlungsbedürftige radiogene Akuttoxizitäten als nach sofortiger Operation (7 % vs. < 1 %; Odds Ratio [OR] = 24,67; *p* < 0,0001; [20]). Bemerkenswert ist, dass trotz der erhöhten Akuttoxizitäten die Rate an postoperativen Komplikationen nach verzögerter TME mit 41 % geringer war als nach sofortiger Operation mit 53 % (OR = 0,61; *p* = 0,001; [20]).

Mit der Frage der chirurgischen Deeskalation nach Radiatio mit 5 × 5 Gy bei frühen Stadien beschäftigte sich die TREC-Studie (Tab. 3). Eingeschlossen wurden Tumoren der Stadien cT1‑2 cN0 M0 mit maximalem Durchmesser von 3 cm. Es wurde zwischen einem Arm mit alleiniger TAR und TME und einem Arm mit Radiatio mit 5 × 5 Gy und anschließender transanal endoskopischer mikroskopischer Resektion (TEM) nach 8 bis 10 Wochen verglichen. Falls im Präparat nach TEM Risikofaktoren wie beispielsweise eine R1-Resektion feststellbar waren, wurde zur TAR mit TME konvertiert. Andernfalls wurden die Patienten engmaschig nachbeobachtet. Ein Organerhalt war nach Radiatio, gefolgt von TAR mit TEM, in 70 % der Fälle möglich bei vergleichbarem krankheitsfreiem Überleben nach 3 Jahren [21]. In Anbetracht der relativ geringen Fallzahl bleibt abzuwarten, wie die Ergebnisse der aktuell laufenden, größeren STAR-TREC-Studie ausfallen, die 2020 in die Phase III fortschreiten konnte [21].

Im Gegensatz zur TREC-Studie beschäftigte sich die RAPIDO-Studie mit dem lokal fortgeschrittenen Rektumkarzinom. Wesentliche Einschlusskriterien sind Tab. 3 zu entnehmen.

**Table 3:** 

Studie	Einschlusskriterien (Auswahl)	Wesentliche Ergebnisse
Stockholm III	Resektabler Primarius, M0	Nach Kurzzeitradiatio kann die chirurgische Resektion bis zu 8 Wochen nach Radiatio erfolgen, ohne das onkologische Ergebnis oder die Rate an postoperativen Komplikationen zu verschlechtern
TREC	cT1‑2 cN0 M0 mit maximalem Durchmesser von 3 cm	In frühen Stadien kann durch eine Kurzzeitradiatio ggf. auf eine transanal endoskopische mikroskopische Resektion deeskaliert werden
RAPIDO	T4a/b, N2 oder vergrößerte laterale Lymphknoten Infiltration der mesorektalen Faszie Extramurale Veneninfiltration	Durch Kurzzeitradiatio lokal fortgeschrittener Stadien, gefolgt von neoadjuvanter Chemotherapie, kommt es seltener zum krankheitsbedingten Therapieversagen, insbesondere durch seltenere Fernmetastasierung. Dies geht auf Kosten einer erhöhten Toxizität
STELLAR	cT3‑4 und/oder N ^+^	Die Kurzzeitradiatio ist der neoadjuvanten Radiochemotherapie, eingebettet in weitere chirurgische und chemotherapeutische Behandlung, nicht unterlegen
PRODIGE-23	cT3/4	Durch das Vorziehen von Teilen der Chemotherapie vor die neoadjuvante Radiochemotherapie kann ein besseres krankheitsfreies Überleben erreicht werden
CAO/ARO/AIO-12	cT3/4	Werden Teile der Chemotherapie zwischen der neoadjuvanten Chemotherapie und der Resektion gegeben, werden höhere Raten an pathologischer Komplettremission erreicht als beim Vorziehen von Teilen der Chemotherapie vor die neoadjuvante Radiochemotherapie
OPRA	cT3/4	Nach Radiochemotherapie und anschließender Chemotherapie kann in bestimmten Fällen auf die Resektion verzichtet werden

Im Standardarm wurde eine simultane, capecitabinhaltige Radiochemotherapie mit einer kumulativen Gesamtdosis von 50,4 Gy mit anschließender TAR und TME nach 8‑wöchigem Intervall appliziert. Im experimentellen Arm wurde mit einer Kurzzeitradiatio von 5 × 5 Gy, gefolgt von einer Chemotherapie innerhalb von 18 Wochen und daran anschließender TAR und TME therapiert. Den primären Endpunkt stellte das krankheitsbedingte Therapieversagen nach 3 Jahren dar. Hierunter verstand man das Auftreten eines lokoregionären Rezidivs, einer R2-Resektion, einer Fernmetastasierung, eines neuen primären kolorektalen Karzinoms oder den therapieassoziierten Tod [22]. Innerhalb von 3 Jahren kam es im experimentellen Arm signifikant seltener zum krankheitsbedingten Therapieversagen (HR = 0,75; *p* = 0,019). Ein Grund hierfür ist insbesondere eine niedrigere Rate an Fernmetastasierungen im Experimentalarm (20,0 %* vs*. 26,8 %; HR = 0,69; *p* = 0,0048; [22]). Die Rate an pathologischen Komplettremissionen hat sich im experimentellen Arm mit 28 % vs. 14 % im Kontrollarm verdoppelt (OR = 2,37; *p* < 0,0001). Die Häufigkeit von Grad-3/-4-Toxizitäten während der neoadjuvanten Therapiephase war im Experimentalarm mit 48 % vs. 25 % erhöht. Die Rate an R0-Resektionen war in beiden Armen mit ca. 90 % vergleichbar [22]. Die Rate an postoperativen Komplikationen ≥ III nach Clavien-Dindo war in beiden Armen ebenfalls vergleichbar. Allerdings war nach Beurteilung der jeweils Operierenden die mesorektale Faszie im Experimentalarm mit 78 % signifikant seltener intakt als im Kontrollarm mit 85 % (*p* = 0,032; [23]).

In der STELLAR-Studie (Tab. 3) wurden ebenfalls nach MRT(Magnetresonanztomographie)-Kriterien fortgeschrittene Tumoren der Stadien cT3‑4 und/oder N ^+^ untersucht. Hier wurde der Kontrollarm – eine neoadjuvante, simultane Radiochemotherapie ad 50 Gy mit Capecitabin, gefolgt von einer TAR mit TME und anschließend 6 Zyklen CAPOX (Capecitabin und Oxaliplatin) – gegen den Experimentalarm mit 5 × 5 Gy, anschließender Gabe von 4 Zyklen CAPOX, TAR plus TME und hernach noch einmal 2 Zyklen CAPOX verglichen. Die Studie war als Nichtunterlegenheitsstudie angelegt und zeigte hinsichtlich ihres primären Endpunktes, des krankheitsfreien Überlebens nach 3 Jahren, die Nichtunterlegenheit des Experimentalarms gegenüber dem Kontrollarm [24].

Zusammenfassend zu den Studien, die eine Kurzzeitradiotherapie mit 5 × 5 Gy untersuchten, lässt sich sagen, dass insbesondere nach dem RAPIDO-Konzept klinisch relevant die Rate an Fernmetastasierungen reduziert war. Gleichzeitig zeigt die STELLAR-Studie nur die Nichtunterlegenheit, kein signifikant besseres krankheitsfreies Überleben. Bei der Abwägung hinsichtlich einer Therapie analog RAPIDO gilt es in der Praxis, insbesondere die Einschlusskriterien der RAPIDO-Studie in Betracht zu ziehen. Außerdem sollte die erhöhte Toxizität der adjuvanten Chemotherapie im Rahmen einer Therapie nach RAPIDO, insbesondere bei älteren und multimorbiden Patienten bei der Therapieentscheidung miteinbezogen werden.

#### Neoadjuvante simultane Radiochemotherapie

Im Rahmen der PRODIGE-23-Studie (Tab. 3) wurde untersucht, inwiefern bei lokal fortgeschrittenen Tumoren (cT3/4) Teile der normalerweise adjuvanten Chemotherapie nach neoadjuvanter Radiochemotherapie und TAR mit TME bereits vor der neoadjuvanten Radiochemotherapie gegeben werden können. Als Standardtherapie wurde hier die neoadjuvante, simultane Radiochemotherapie ad 50,4 Gy und simultane Gabe von Capecitabin, gefolgt von TAR mit TME und adjuvanter Chemotherapie, definiert. Im experimentellen Arm wurden vor der neoadjuvanten Radiochemotherapie Teile der Chemotherapie appliziert, nach TME wurde die restliche Chemotherapie gegeben. Es zeigte sich ein signifikant besseres krankheitsfreies Überleben nach 3 Jahren im experimentellen Arm mit 76 % gegenüber dem Standardarm mit 69 % (stratifizierte HR = 0,69; *p* = 0,034). Darüber hinaus konnte durch das Vorziehen der Chemotherapie ein signifikant besseres metastasenfreies Überleben nach 3 Jahren gezeigt werden (79 % vs. 72 %, stratifizierte HR = 0,64; *p* = 0,017; [25]).

In der randomisierten Phase-II-Studie CAO/ARO/AIO-12 (Tab. 3) wurde dieser Gedanke zu lokal fortgeschrittenen Rektumkarzinomen weitergeführt und untersucht, in welcher Reihenfolge die neoadjuvanten Therapien appliziert werden sollen. Hierzu wurde im Standardarm mit der Chemotherapie begonnen, gefolgt von der simultanen Radiochemotherapie ad 50,4 Gy mit Oxaliplatin und 5‑Fluorouracil (5-FU). Im Experimentalarm wurde zuerst die simultane Radiochemotherapie und anschließend die Chemotherapie appliziert. In beiden Armen erfolgte im Anschluss die TAR/TME. Der primäre Endpunkt war die pathologische Komplettremission. Es zeigte sich, dass bei simultaner neoadjuvanter Radiochemotherapie und daran anschließender Chemotherapie eine höhere Rate an pathologischen Komplettremissionen erreicht wurde (25 % vs. 17 %; *p* < 0,001). Gleichzeitig ging die längere Wartezeit nach Radiochemotherapie nicht mit einer erhöhten chirurgischen Morbidität einher [26]. Die unlängst publizierten Langzeitdaten der Studie konnten mit 73 % in beiden Armen keinen signifikanten Unterschied hinsichtlich des 3‑jährigen krankheitsfreien Überlebens zeigen. Außerdem ging die verbesserte Rate an pathologischen Komplettremissionen nicht zulasten von Lokalrezidiven, einer Fernmetastasierung, der Toxizität, der Lebensqualität und der Stuhlinkontinenz [27].

Der nächste Schritt in dieser Entwicklung bei der Therapie des lokal fortgeschrittenen Rektumkarzinoms könnte der Versuch des Organerhalts sein. Dies wurde in der OPRA-Studie (Tab. 3), einer prospektiv randomisierten Phase-II-Studie, untersucht. In einem Arm wurde eine Induktionschemotherapie mit anschließender simultaner fluorouracilhaltiger Radiochemotherapie ad 50 Gy (INCT-CRT) appliziert, im anderen Arm war diese Sequenz vertauscht (CRT-CNCT). Hernach fand nach ca. 8 Wochen ein Restaging statt. Falls eine klinische Komplettremission vorlag, wurde anstelle der TAR mit TME eine „Watch-and-wait“-Strategie vorgeschlagen. Als Kontrollgruppe dienten historische Daten eines Kollektivs mit neoadjuvanter Radiochemotherapie, TAR mit TME und adjuvanter Chemotherapie. Der primäre Endpunkt war das krankheitsfreie Überleben nach 3 Jahren, als sekundärer Endpunkt wurde das TAR/TME-freie Überleben untersucht. Hinsichtlich des krankheitsfreien Überlebens nach 3 Jahren zeigte sich mit jeweils 76 % kein signifikanter Unterschied zwischen den Behandlungsregimen untereinander, aber auch nicht zur historischen Kontrolle (75 %). Es zeigte sich allerdings ein signifikant besseres TAR/TME-freies Überleben in der Gruppe mit CRT-CNCT mit 53 % vs. 41 % in der INCT-CRT-Gruppe. In beiden Armen war die Rate an klinischer Komplettremission vergleichbar. Die Rate an Wiederwachstum war in der INCT-CRT aber mit 40 % höher als nach CRT-CNCT (27 %), sodass hierin ein möglicher Grund für das bessere TAR/TME-freie Überleben liegen könnte [28]. Für die klinische Praxis zeigen sich hier Hinweise, dass bei der Therapie des lokal fortgeschrittenen Rektumkarzinoms erst eine Radiochemotherapie und als anschließende Modalität eine Chemotherapie empfehlenswert ist. Hierbei spricht man häufig von einer „totalen neoadjuvanten Therapie“, die, obgleich noch nicht vollständig in den Leitlinien verankert, zunehmend häufig zur Anwendung kommt. Zur Feinabstimmung der totalen neoadjuvanten Therapie laufen aktuell zahlreiche Studien. Beispielhaft sei hier die ACO/ARO/AIO-18.1, die die Kurzzeitradiatio mit der Radiochemotherapie als radioonkologischen Baustein einer totalen neoadjuvanten Therapie vergleicht, genannt. Das Studienende wird für 2025 erwartet.

Generell spielt im Rahmen des Stagings des Rektumkarzinoms die MRT eine wichtige Rolle. In die Entscheidung hinsichtlich einer neoadjuvanten Radiotherapie fließen häufig die NICE(National Institute for Health and Care Excellence)-Kriterien ein. Analog NICE werden alle Tumoren außer T1‑2 N0 als hochriskant eingestuft und eine neoadjuvante Radiotherapie empfohlen. In einer retrospektiven Kohortenstudie mit insgesamt 378 Patienten wurden zwei Gruppen von operierten, aber nichtradiierten Patienten verglichen. Eine Gruppe wurde analog den NICE-Kriterien gefasst, für eine zweite Gruppe wurden lediglich folgende MRT-basierte Kriterien für einen hochriskanten Tumor formuliert: extramurale Veneninfiltration, Befall des zirkumferenziellen Resektionsrandes und Tumorabsiedlungen innerhalb des Mesorektums. Dabei wurden nach NICE 66 % der Fälle als hochriskant kategorisiert, nach MRT-Kriterien jedoch lediglich 32 %. Nach multivariabler Analyse zeigte sich, dass die NICE-Kriterien weder mit dem krankheitsfreien Überleben noch mit dem Gesamtüberleben assoziiert waren. Die MRT-Kriterien waren jedoch sowohl hinsichtlich des krankheitsfreien Überlebens als auch des Gesamtüberlebens prädiktiv. Möglicherweise findet bei alleiniger Verwendung der 2020 NICE-Kriterien also eine Übertherapie bei einem Teil der Patienten statt [29]. Dies unterstreicht die Bedeutung der MRT im Rahmen des Rektumkarzinomstagings.

#### Schlussfolgerung

In den UICC(Union internationale contre le cancer)-Stadien II und III soll laut aktueller S3-Leitlinie i. d. R. eine neoadjuvante Radiochemotherapie oder eine Kurzzeitradiatio durchgeführt werden. Dabei wird häufig der neoadjuvanten Radiochemotherapie der Vorzug gegeben, wenn ein Downsizing des Tumors gewünscht ist. Im Lichte der aktuellen Studienlage lässt sich der Trend zur totalen neoadjuvanten Therapie mit dem Verzicht auf die Resektion ausmachen. Hierfür kommt insbesondere ein Patientenkollektiv in gutem Allgemeinzustand infrage, da die Therapie analog RAPIDO häufig mit intensiveren Nebenwirkungen einhergeht. Ein längeres Zuwarten nach Kurzzeitradiatio scheint nicht mit einer erhöhten chirurgischen Komplikationsrate einherzugehen.

### Analkarzinom

#### Epidemiologie

Mit einer Inzidenz von 1 bis 2 pro 100.000 zählt das Analkarzinom zu den seltenen Karzinomen. Die Inzidenz des Analkarzinoms ist in den letzten Jahren jedoch um durchschnittlich 4,6 % pro Jahr stetig gestiegen [30]. Histologisch handelt es sich meist um Plattenepithelkarzinome. Zwischen 80 und 90 % der Fälle an Analkarzinomen sind HPV(humane Papillomviren)-assoziiert [31].

#### Staging

Beim Staging des Analkarzinoms spielt insbesondere die MRT eine wichtige Rolle. Aus radioonkologischer Sicht kann aber auch eine PET/Computertomographie(CT) das Staging sinnvoll ergänzen. So konnte gezeigt werden, dass die Zuhilfenahme eines PET/CT im Rahmen der Zielvolumendefinition in ca. 20–30 % der Fälle zu einem veränderten Zielgebiet führt [32]. Häufig wird die Dosis an inguinalen Lymphknoten bei sowohl im MRT als auch im PET/CT unauffälligen inguinalen Lymphknoten analog auf 30,6–36 Gy reduziert [33].

Aufgrund der Tumorlage gehen primäre chirurgische Therapieoptionen i. d. R. mit der Anlage eines dauerhaften Anus praeter einher. Ziel der Therapie ist neben der Tumorkontrolle ein bestmöglicher Erhalt der Lebensqualität.

#### Primär definitive Radiochemotherapie

**Figure 3:**
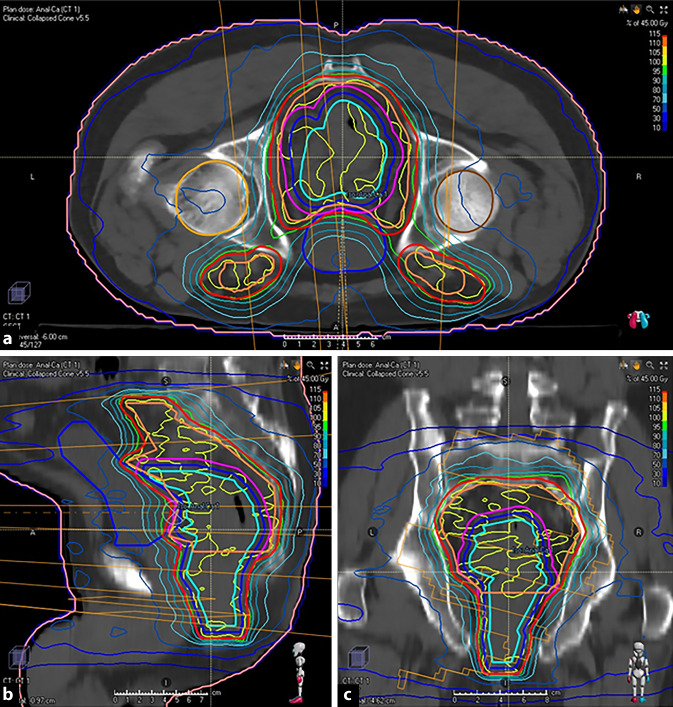

Bereits Nigro et al. etablierten die simultane Radiochemotherapie mit 5‑FU und Mitomycin, die heute als Therapie der Wahl gilt. Sie konnten zeigen, dass nach diesem Vorgehen in 86 % der Fälle histologisch kein Tumor mehr nachweisbar war ([34, 35]; Abb. 3). Nachfolgende Studiengenerationen zeigten, dass die simultane Radiochemotherapie mit 5‑FU und Mitomycin sowohl der alleinigen Radiatio als auch der kombinierten Radiochemotherapie mit 5‑FU ohne Mitomycin hinsichtlich der lokalen Kontrolle, des kolostomiefreien Überlebens und des Gesamtüberlebens überlegen ist [36, 37]. In weiteren Fallserien und Phase-I- und Phase-II-Studien konnte kein signifikanter Vorteil durch die Hinzunahme von Cetuximab gezeigt werden [38, 39].

Hinsichtlich einer Induktionschemotherapie vor Radiochemotherapie soll an dieser Stelle die ACCORD-03-Studie (Tab. 4), eine Phase-III-Studie, betrachtet werden. In die ACCORD-03-Studie wurden Analkarzinome mit einer Tumorgröße ≥ 40 mm oder mit lymphonodaler Metastasierung eingeschlossen. Anstelle von Mitomycin wurde im Rahmen dieser Studie Cisplatin mit 5‑FU kombiniert. Im Kontrollarm wurde eine Radiochemotherapie mit simultaner Gabe von Cisplatin und 5‑FU durchgeführt. Im Experimentalarm wurden zuvor noch 2 Zyklen Induktionschemotherapie mit Cisplatin und 5‑FU appliziert. Hierbei zeigte sich sowohl hinsichtlich des primären Endpunktes des kolostomiefreien Überlebens als auch hinsichtlich der Lokalkontrolle, des tumorfreien Überlebens und des krankheitsspezifischen Überlebens kein signifikanter Unterschied zwischen den Untersuchungsarmen [40, 41].

Auch die RTOG-98-11-Studie (Tab. 4), in die alle Tumoren zwischen cT2‑4 und Lymphknotenstatus cN0‑3 eingeschlossen wurden, konnte hinsichtlich der Induktionschemotherapie vor primär definitiv intendierter Radiochemotherapie keinen Vorteil zugunsten der Induktionschemotherapie nachweisen. Es zeigte sich sogar ein signifikanter Vorteil der Radiochemotherapie mit Mitomycin und 5‑FU gegenüber einem Therapieregime mit vorheriger Induktionschemotherapie im Blick auf die klinische Komplettremission, das krankheitsfreie Überleben und das Gesamtüberleben [33].

**Table 4:** 

Studie	Einschlusskriterien (Auswahl)	Wesentliche Ergebnisse
ACCORD-03	Tumorgröße ≥ 40 mm oder N ^+^	Eine Induktionschemotherapie vor primär definitiv intendierter Radiochemotherapie zeigt keinen signifikanten Vorteil
RTOG-98-11	cT2‑4, cN0‑3	Eine Induktionschemotherapie vor primär definitiv intendierter Radiochemotherapie zeigt keinen signifikanten Vorteil
ACT I	Klinisches Staging, selten radiologisches Staging, M0	Die simultane Radiochemotherapie ist der alleinigen Radiatio überlegen
ACT-II	Alle T, alle N, M0	Kein signifikanter Vorteil hinsichtlich des progressionsfreien Überlebens oder des Gesamtüberlebens durch eine konsolidierende Chemotherapie

Die ACT-II-Studie (Tab. 4), ebenfalls eine Phase-III-Studie, beschäftigte sich mit der Frage nach einer konsolidierenden Chemotherapie nach primär definitiver Radiochemotherapie. Dabei wurden alle Tumorstadien ohne Fernmetastasierung eingeschlossen. Weder hinsichtlich des primären Endpunktes, des progressionsfreien Überlebens, noch des Gesamtüberlebens zeigte sich ein signifikanter Vorteil durch die konsolidierende Chemotherapie [42]. Eine Sekundäranalyse der Daten der ACT-II-Studie befasste sich mit der Frage nach der Beurteilung des Therapieansprechens nach primär definitiver Radiochemotherapie. Hierbei wurde nach 11, 18 und 26 Wochen nach Beginn der Radiochemotherapie das Ansprechen mittels rektal-digitaler Untersuchung, nach 26 Wochen ergänzt um ein CT, evaluiert. Hierbei zeigte ich nach 11 Wochen bei 64 % der Patienten, nach 18 Wochen bei 80 % und nach 26 bei 85 % eine klinische Komplettremission. Das 5‑Jahres-Gesamtüberleben war bei Patienten mit klinischer Komplettremission mit 87 % signifikant besser als bei Patienten ohne klinischer Komplettremission mit lediglich 48 % [43].

#### Schlussfolgerung

Zusammenfassend zeigt sich, dass die primär definitive Radiochemotherapie nach wie vor der Standard in der Therapie des Analkarzinoms bleibt. Dies zeigt sich auch in der aktuellen S3-Leitlinie. Lediglich das Analrandkarzinom < 2 cm soll lokal exzidiert werden. In allen anderen Stadien ohne Fernmetastasierung sowie beim Analkanalkarzinom < 2 cm soll oder sollte die primär definitive Radiochemotherapie zum Zug kommen. Aus Sicht des Radioonkologen ist ein PET/CT zwar nicht zwingend erforderlich, zur Zielvolumendefinition jedoch oft wünschenswert.

### Lebertumoren – Strahlentherapie zur lokalen Ablation?

#### Epidemiologie

Die Leber ist das Organ, welches mit am häufigsten von Metastasen solider Tumoren befallen wird. Insbesondere bei kolorektalen Karzinom treten in ca. 50 % der Fälle hepatische Metastasen auf. Eine chirurgische Resektion ist in etwa 25 % der Fälle möglich und ist im Falle einer kurativ intendierten Resektion mit einer 5‑Jahres-Gesamtüberlebensrate von 40–50 % verbunden [44]. In der Mehrzahl der Fälle liegen jedoch zum Zeitpunkt der Diagnosestellung bereits multiple oder solitäre inoperable Lebermetastasen vor. Bei solitärer oder auf die Leber beschränkter (oligometastatischer) nichtoperabler Metastasierung sollten lokal ablative Verfahren angewandt werden.

#### Stereotaxie als Therapieoption

**Figure 4:**
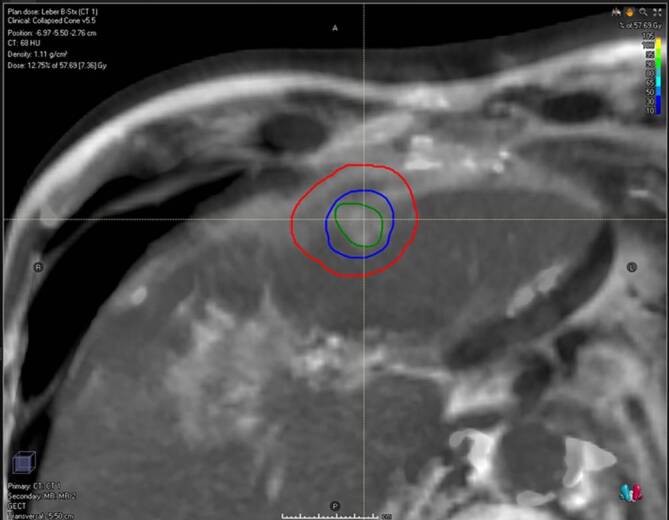

Mit der erstmalig 1995 angewendeten [45] extrakraniellen stereotaktischen Radiotherapie verfügt die Radioonkologie über ein nichtinvasives Hochpräzisionsverfahren zur lokalen Ablation metastatischer Läsionen, welches eine gute Lokalkontrolle bei sehr guter Verträglichkeit und geringen Nebenwirkungen bietet [46]. Das Verfahren wurde in den 1990er-Jahren in Deutschland etabliert und ist flächendeckend verfügbar ([47]; Abb. 4).

Für diese Form der Bestrahlung wird insbesondere die IMRT-Technik („intensity modulated radiotherapy“) verwendet [48]. In der Literatur liegt das 2‑Jahres-Überleben bei Anwendung dieser Therapie zwischen 32 % und 83 % bei der Behandlung kolorektal bedingter Metastasen [49, 50]. Studien mit 5‑Jahres-Überlebensdaten mit größeren Patientenkohorten oder randomisierte Studien, die die Stereotaxie mit einer Resektion oder Thermoablation vergleichen, liegen für kolorektale Lebermetastasen aktuell nicht vor.

Multizentrische „Patterns-of-care“-Analysen zeigen eine mediane 1‑Jahres-Lokalkontrollrate von 84 % (Streubreite: 81–100 %) bei geringen Toxizitäten.

Unter ausreichender Schonung des gesunden Lebergewebes können 1 bis 3 Metastasen (≤ 6 cm Durchmesser) simultan bestrahlt werden. Unter Anwendung moderner Rotationstechnicken („volumetric intensity modulated arc therapy“, VMAT ) ist auch die Bestrahlung einer größeren Zahl von Metastasen möglich, insofern ein Lebervolumen von etwa 800 ml geschont werden kann. Zunehmend wird auch die stereotaktische Bestrahlung lebereigener Tumoren, wie z. B. das hepatozelluläre Karzinom (HCC), durchgeführt [46].

Nach der diagnostischen CT- und MRT-Bildgebung im Rahmen der Bestrahlungsplanung erfolgt zur exakten Lokalisation des Zielgebiets eine Fixierung in einem „Körper-Stereotaxis-Rahmen“. Außerdem kommt das sog. „Atem-Gating“ (Berücksichtigung der Atembeweglichkeit des Organs) zum Einsatz [46]. Durch exakte Verifikation und Reproduktion von Patienten- und Tumorposition können hohe Einzeldosen appliziert werden. Ein häufig angewendetes Konzept, sowohl für Metastasen als auch lebereigene Tumoren, ist die Applikation von 3 × 12,5 Gy auf die 65 % umschließende Isodose, über eine Woche verteilt, wodurch die Nebenwirkungen reduziert werden sollen [46].

#### Schlussfolgerung

Nach Ausschöpfen alternativer lokal ablativer Verfahren steht sowohl zur Therapie lebereigener Tumoren als auch von Lebermetastasen mit der Leberstereotaxie ein probates Mittel zur Verfügung.

## Resümee

Die Strahlentherapie ist ein integraler Bestandteil in einer Vielzahl moderner und etablierter multimodaler Tumortherapiekonzepte, sowohl in kurativer als auch in palliativer Therapieintention, wie das hinsichtlich allgemein- und viszeralchirurgischer Relevanz insbesondere für das Rektum‑, Ösophagus- und Analkarzinom sowie für Lebermetastasen von Bedeutung ist.

Die enge interdisziplinäre Zusammenarbeit der viszeralmedizinischen Fächer im Rahmen der Tumortherapie erscheint essenziell für einen bestmöglichen antitumorösen Therapieeffekt der anvertrauten Patienten.
