# Erratum zu: Was muss der Allgemein- und Viszeralchirurg von der onkologisch ausgerichteten Strahlentherapie wissen?

**DOI:** 10.1007/s00104-023-01863-4

**Published:** 2023-04-17

**Authors:** Jörg Andreas Müller, Simon Trommer, Frank Meyer, Katharina Lampe, Roland S. Croner, Dirk Vordermark, Daniel Medenwald

**Affiliations:** 1 Universitätsklinik und Poliklinik für Strahlentherapie, Universitätsklinikum Halle (Saale), Halle (Saale), Deutschland; 2 Klinik für Allgemein‑, Viszeral‑, Gefäß- und Transplantationschirurgie, Universitätsklinikum Magdeburg A. ö. R., Leipziger Str. 44, 39120 Magdeburg, Deutschland; 3 Klinik für Strahlentherapie, Universitätsklinikum Magdeburg A. ö. R., Magdeburg, Deutschland

**Erratum zu:**

**Chirurgie 2023**

10.1007/s00104-023-01820-1

In diesem Artikel fehlten die Adressdaten bzw. die Daten zur institutionellen Zugehörigkeit für die Autoren Jörg Andreas Müller, Simon Trommer, Katharina Lampe, Roland S. Croner, Dirk Vordermark und Daniel Medenwald. Bitte beachten Sie die oben genannten Adressdaten. Der Originalbeitrag wurde korrigiert.

